# Supplementary material for: When Corticospinal Inhibition Favors an Efficient Motor Response
Source: Biology (Basel). 2023 Feb 20;12(2):332. doi: 10.3390/biology12020332 (PMC9953307; doi:10.3390/biology12020332)
Supplement: Supplementary file 1 [file biology-12-00332-s001.zip › biology-2029674-supplementary.pdf]

Supplementary Materials for:

Corticospinal inhibition in dyadic motor contexts favors an efficient motor response

Sonia Betti [1,2]*, Giovanni Zani [3], Silvia Guerra [2], Umberto Granziol [2], Umberto Castiello [2,4], Chiara Begliomini [2,5] and Luisa Sartori [2,5]

[1] Department of Psychology, Centre for Studies and Research in Cognitive Neuroscience, University of Bologna, viale Rasi e Spinelli 176, 47521, Cesena, Italy
[2] Department of General Psychology, University of Padova, via Venezia 8, 35131, Padova, Italy
[3] School of Psychology, Victoria University of Wellington, Kelburn Parade 20, 6012, Wellington, New Zealand
[4] Padova Center for Network Medicine, University of Padova, via Francesco Marzolo 8, 35131, Padova, Italy
[5] Padova Neuroscience Center, via Giuseppe Orus 2, 35131, University of Padova, Padova, Italy
* Correspondence: sonia.betti@unipd.it

1. Validation Study

To validate the naturalistic paradigm and the action sequences adopted in the main experiment, a preliminary study was conducted. The participants had to watch video clips and fill out a questionnaire after each video presentation. In particular, the video clips showed a co-experimenter in a third-person perspective performing a gesture toward an actor showed in a first-person perspective. Crucially, the video was shot so that only the actor's right hand was visible in the bottom right corner, as if the observer was actually participating in the interaction (Figure S1).

1.1 Materials and methods

1.1.1 Participants

Fifty naïve volunteers (36 women and 14 men, between 18 and 74 years old, mean age 28.41 ± 12.93 years) participated in the experiment. All participants were right-handed, as assessed with the Edinburgh Handedness Inventory [1], with normal or corrected-to-normal visual acuity. Data from one participant were excluded from the analysis due to technical problems; then, 49 participants were included in the analysis. A right-handed non-professional actor (female, 30 years old) was recruited for the videoclip recordings.

1.1.2 Experimental stimuli

Four video clips were adopted as experimental stimuli (see Suppl. Fig. 1):

1. Interactive request, precision grip (PG; i.e., a thumb's opposition to the index finger) execution: the co-experimenter grasps a sugar spoon, takes some sugar from a sugar bowl and then stretches out her arm toward an out-of-reach coffee cup containing a teaspoon, as if to pour the sugar in it. Then, a right hand, visible in the bottom right corner of the screen, grasps the teaspoon with a PG and stirs the coffee (Figure S1A).

2. Non-Interactive action, PG execution: the co-experimenter grasps the sugar spoon, takes some sugar from the sugar bowl, and then returns to the initial position. The right hand in the bottom right corner grasps the teaspoon with a PG and stirs the coffee (Figure S1B).

3. Interactive request, whole hand grasp (WHG; i.e., the opposition of the fingers to the palm) execution: the co-experimenter grasps the sugar spoon, takes some sugar from the sugar bowl, and then stretches out her arm toward the out-of-reach coffee cup containing the teaspoon, as if to pour

some sugar in it. The right hand, visible in the bottom right corner, grasps the cup with a WHG and lifts it up (Figure S1C).

1. Non-Interactive action, WHG execution: the co-experimenter grasps the sugar spoon, takes some sugar from the sugar bowl, and then returns to the initial position. The right hand in the bottom right corner grasps the cup with a WHG and lifts it up to the co-experimenter (Figure S1D).

Each video lasted 10 s (25 frames per second, resolution 1440 x 1080 pixels, color depth 32 bits).

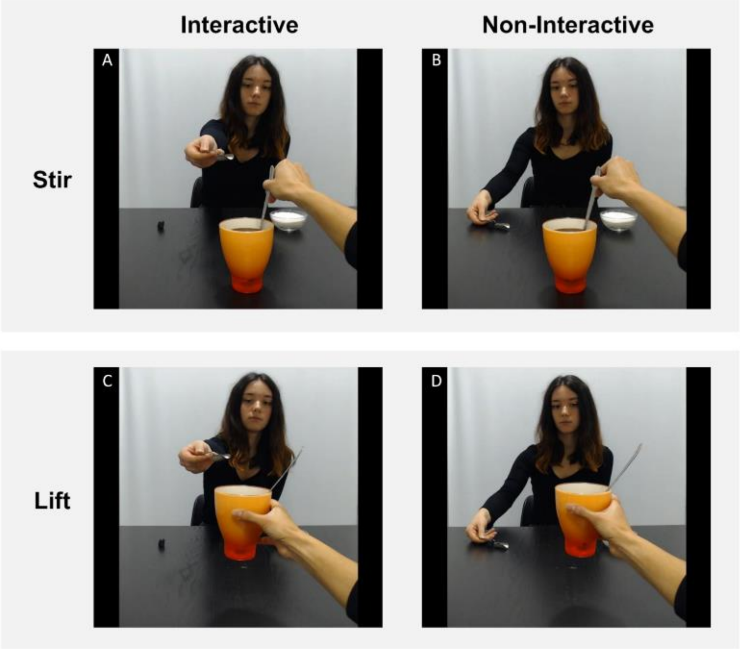

Figure S1. Video stimuli adopted in the preliminary validation study. The video clips showed a co-experimenter in a third-person perspective performing an action that could be either Interactive (**A**, **C**) or Non-interactive (**B**, **D**). Eventually, another agent, whose hand is visible from a first-person perspective, performed another action, that is, a PG to stir the coffee (**A**, **B**) or a WHG to lift the coffee cup (**C**, **D**).

2.1.3 Procedure

Participants were told to carefully watch four videos and answer three questions about the stimuli at the end of each video presentation. In particular, they were instructed to express their ratings on a five-point Likert scale (ranging from “not at all” to “very much”). The order of the videos was randomized. Hereafter, the adopted items translated from Italian: Q1) “Is the girl’s action interactive?”; Q2) “Do I feel involved in the action?”; Q3) “Does the actor in the first person view correctly reply to the girl’s gesture?”. At the end of the questionnaire, participants were also asked what gesture between stirring or lifting they considered more socially appropriate for the Interactive

conditions. These sentences were adopted to quantify the subjective experience of involvement experienced by each participant and to assess whether the experimental stimuli were effective in modulating perceived social appropriateness (for a similar approach, see [2]).

2.1.4 Data analysis

Statistical analyses were performed using generalized mixed effects models (GLMMs). For each set of responses to each item (Q1, Q2, Q3), a separate GLMM was set. The differences in scores between both PG versus WHG and Interactive versus Non-Interactive conditions were tested as fixed effect. The interaction between the two predictors was estimated. Subjects were set as random factor (i.e., random-intercept models were used).

All statistical analyses were performed using the computing environment R (R Core Team, 2012). The effect size of each GLMM's predictor was estimated by using the *effectsize* and *emmeans*.

2.2 Results and Discussion

The results of GLMMS showed a significant difference among Interactive and Non-Interactive conditions. In general, Interactive videos obtained higher scores for all items (Q1: β= -0.51; $p < .001$; *d*= -0.58; Q2: β= -1.10; $p< .001$; *d*=-1.16; Q3: β= -0.41; $p = .02$; *d*= -0.33), suggesting an increased perceived interactivity and involvement when an interactive request was shown. For both Q1 and Q2 items, this main effect was independent from the type of action observed in the first-person perspective (i.e., a PG or a WHG), as suggested by the non-statistically significant interactions (Q1: β= -0.07; $p = .68$; *d*= -0.06; Q2: β= -0.01; $p = .96$; $d < .01$). We did not find a main difference among videos showing PG and WHG for both items (Q1: β= -0.51; $p < .001$; *d*= -0.58; Q2: β= -0.51; $p < .001$; *d*= -0.58). Watching the actor in the first-person view lifting the cup (WHG) in the Interactive condition, compared to the Non-Interactive conditions, increased the scoring for both items Q1 (β= -0.51; $p < .001$; *d*= -0.58) and Q2 (β= -1.10; $p < .001$; *d*= -1.16). The same was obtained for the video showing the actor stirring the spoon (PG) in the Interactive condition, compared to the Non-Interactive conditions, for items Q1 and Q2 (β= -0.58; $p < .001$; *d*= -0.64 and β= -1.11; $p < .001$; *d*= -1.09, respectively).

Regarding Q3, we observed a main effect of the type of action, with higher scorings for WHG than PG actions (β= 1.12; $p < .001$; *d*= 0.67). We also observed a significant interaction (β= -0.95; $p < .01$; *d*= -0.46), with higher scoring for the Interactive compared to the Non-Interactive condition only for videos involving the WHG (β= -1.36; $p < .001$; *d*= -0.83). For PGs, the difference between Interactive

and Non-interactive videos was not statistically significant (β= -0.41; p = .11; *d*= -0.33). This seems to suggest that observing the girl stretching out her arm toward the out-of-reach coffee cup was perceived as interactive and engaging, but the associated gesture of stirring the coffee was not considered the most appropriate. In fact, when asked what gesture they considered more socially appropriate for the Interactive conditions, 73% of participants replied "lifting the cup" and 27% "stirring the coffee".

References

1. Oldfield, R.C. The Assessment and Analysis of Handedness: The Edinburgh Inventory. *Neuropsychologia* **1971**, *9*, 97–113, doi:10.1016/0028-3932(71)90067-4.
2. Betti, S.; Zani, G.; Granziol, U.; Guerra, S.; Castiello, U.; Sartori, L. Look At Me: Early Gaze Engagement Enhances Corticospinal Excitability During Action Observation. *Front. Psychol.* **2018**, *9*, 1408, doi:10.3389/fpsyg.2018.01408.
